# Complaints of daytime sleepiness, insomnia, hypnotic use, and risk of dementia: a prospective cohort study in the elderly

**DOI:** 10.1186/s13195-021-00952-y

**Published:** 2022-01-20

**Authors:** Clémence Cavaillès, Claudine Berr, Catherine Helmer, Audrey Gabelle, Isabelle Jaussent, Yves Dauvilliers

**Affiliations:** 1 Institute for Neurosciences of Montpellier INM, INSERM Montpellier, University of Montpellier, Montpellier, France; 2 INSERM, Bordeaux Population Health Research Center, UMR1219, University of Bordeaux, Bordeaux, France; 3 Memory Research and Resources Center, Department of Neurology, CHU Montpellier, Montpellier, France; 4 National Reference Centre for Orphan Diseases, Narcolepsy- Rare Hypersomnias, Sleep Unit, Department of Neurology, CHU Montpellier, Service de Neurologie, Hôpital Gui-de-Chauliac, 80 avenue Augustin Fliche, 34295 Montpellier cedex 5, France

**Keywords:** Sleepiness, Hypnotic use, Insomnia complaints, Dementia, Alzheimer, Elderly, Cohort studies, Epidemiology

## Abstract

**Background:**

Sleep disturbances are common in elderly and occur frequently in dementia. The impact of excessive daytime sleepiness (EDS), insomnia complaints, sleep quality, and hypnotics on the risk of all-cause dementia, Alzheimer disease (AD), and dementia with vascular component (DVC) remains unclear, as does the association between sleep profile and plasma β-amyloid levels.

**Methods:**

Analyses were carried out on 6851 participants aged 65 years and over randomly recruited from three French cities and free of dementia at baseline. A structured interview and self-questionnaire assessed sleep complaints (EDS, insomnia complaints, sleep quality) and medications at baseline. Incident cases of dementia were diagnosed systematically over a 12-year period. Multivariate Cox models were used to estimate the risk of dementia associated with the sleep complaints considered individually and globally. Plasma β-amyloid levels were measured by an xMAP-based assay technology in 984 subjects.

**Results:**

After adjustment for socio-demographic characteristics, lifestyle, APOE-ε4, cardiovascular factors, and depressive status, EDS had a higher risk of all-cause dementia (HR = 1.21; 95%CI = [1.01–1.46]) and DVC (HR = 1.58; 95%CI = [1.07–2.32]) but not AD. Persistent use of hypnotics increased the risk for all-cause dementia, specifically AD (HR = 1.28; 95%CI = [1.04–1.58]), but not DVC. No association was found for insomnia complaints and sleep quality taken as individual factors or combined with EDS on the risk of dementia. No association was found between β-amyloid, sleep complaints, and incident dementia.

**Conclusions:**

The results suggest a deleterious role of EDS and hypnotics on dementia. Further studies are required to elucidate the mechanisms involved in these associations and whether its management can prevent the risk of dementia.

## Introduction

The burden of dementia is important and continues to increase [1]. Since no pharmacological treatment has shown to be effective, the need to prevent dementia is a major public health issue. An increasing attention has been paid to sleep recently. Aging in the general adult population is associated with an increase of sleep problems particularly insomnia and excessive daytime sleepiness (EDS) which have been associated with a higher risk of all-cause cognitive decline or dementia [2–11]. However, large heterogeneities in the results were observed and could be attributed to multifactorial nature and cause of sleep disturbances, differences in sleep and cognitive assessments, use of hypnotics, study design, duration of follow-up, and sample size [2, 3, 5, 11–13]. A recent large-scale study of only men showed that sleep problems defined as difficulties falling asleep, waking up in the early hours, or just taking sleeping pills were associated with dementia incidence, specifically Alzheimer’ disease (AD) [8]. However, the specific role of these three complaints was not studied, and the impact of EDS was not evaluated. Furthermore, the impact of sleep disturbances may depend on the etiologies of dementia associated with different pathophysiologies. Dementia and particularly AD is mainly characterized by excessive production and/or cleavage of amyloid precursor protein and by intra-neuronal hyperphosphorylated and aggregated tau proteins with altered brain clearance. In contrast, although with possible overlap with AD, dementia with vascular component (DVC) is primarily associated with comorbid cardiovascular conditions, as well as hypoxia, inflammation, and oxidative stress. Yet, few studies have investigated the associations between sleep disturbances and the type of dementia and showed that insomnia was often predictive of all-cause dementia and AD [7, 8, 10], and EDS of DVC [3].

As demographic and clinical characteristics may be associated with both sleep problems and dementia, it remains important to determine whether certain populations (e.g., old age, women, carriers of apolipoprotein e-ε4 (APOE-ε4), cardiometabolic diseases, or depression) may modify the associations between sleep complaints and dementia risk. It is also unknown whether there is a synergistic effect of sleep problems on the risk of cognitive disorders. A more complete assessment of sleep problems and their severity may clarify their potential additive roles on the risk of dementia.

We thus evaluated the association between the use of hypnotics, sleep complaints (EDS, insomnia complaints, and sleep quality) analyzed separately as individual dimensions and combined together in an overall assessment, and the risk of dementia (all-cause, AD, and DVC) over 12 years in the elderly. We also studied if specific populations modify the association between sleep problems, hypnotic intake, and dementia risk and deciphered the relationship between sleep profile and plasma β-amyloid concentrations.

## Methods

### Study population

This study was part of the Three-City Study [14], a longitudinal cohort conducted in three French cities: Bordeaux (*n* = 2104), Dijon (*n* = 4931), and Montpellier (*n* = 2259). Participants were non-institutionalized subjects, aged 65 years or over, and registered on the electoral rolls. Its main objective was to estimate the risk of dementia attributable to vascular factors. Overall, 9294 participants were recruited from the electoral rolls between 1999 and 2001. The participants were interviewed and underwent clinical examinations at baseline and after 2, 4, 8, 10, and 12 years.

### Dementia diagnosis

Dementia was screened and diagnosed at baseline and at each follow-up visit according to DSM-IV revised criteria and further validated independently by a national panel of neurologists. The onset of dementia was estimated at the midpoint between diagnosis and the prior examination without dementia. Etiology was based on all available information (e.g., neuropsychological test, daily activities, neurological examination, and hospitalization records), on the National Institute of Neurological and Communicative Disorders and Stroke–Alzheimer’s Disease and Related Disorders Association criteria for Alzheimer disease (AD) and, the National Institute of Neurological Disorders and Stroke–Association Internationale pour la Recherche et l’Enseignement en Neurosciences criteria for vascular dementia [15, 16]. The causes of dementia were divided into three types: AD, DVC (including pure vascular dementia and mixed dementia due to a small number of cases), and other dementias (including dementia with Lewy bodies, Parkinson’s dementia, fronto-temporal dementia [17–20].

### Sleep complaints and medication

Current sleep problems were self-reported at baseline by the completion of a specific questionnaire including 5 items: “How would you assess your sleep?” (sleep quality (SQ)); “Do you feel very sleepy during the day?” (EDS); “Do you have any difficulty in falling sleep?” (difficulty in initiating sleep (DIS)); “Do you wake-up during the night?” (difficulty maintaining sleep (DMS)); “Do you often wake-up early in the morning without being able to go back to sleep?” (early morning awakening (EMA)). The questions were answered on a 4-point Likert scale (0 = never, 1 = rarely, 2 = frequently, 3 = often) except for SQ rated on 3-point (0 = good, 1 = average, 2 = bad). Sleep complaints were first examined as individual dimensions.

The presence of EDS, DIS, DMS, and EMA was defined as reporting the related complaint “frequently” or “often,” and a poor SQ as reporting “average” or “poor” SQ to allow a sufficient number of dementia cases in each sleep category.

As a second step, we proposed to combine together the sleep complaints (EDS, insomnia complaints, and sleep quality) in a clinical sleep severity (CSS) score to estimate their potential additive role on the risk of dementia. CSS score was defined by the sum of the 5-item responses. To standardize the response choice, the SQ item was recoded as 0 = good, 1.5 = average, and 3 = bad. A total score ranged from 0 to 15 with a higher score indicated more severe sleep problems. It was studied as a continuous variable but also as a categorical variable according to the tertiles of the score distribution in the whole sample.

At each visit, the use of prescribed drugs during the preceding month was checked by the interviewer. Current use of medication was based the World Health Organization Anatomical Therapeutic Chemical (ATC) classification system. Hypnotic intake was defined as taking at least one benzodiazepines (BZD) or BZD-like compounds (zolpidem, zopiclone), or miscellaneous medications (including barbiturates, antihistaminics, and other pharmacological categories such as neuroleptics and sedative antidepressants (e.g., doxepin, mirtazapine, trazodone)) during the preceding month.

### Other variables measured at inclusion

Standardized evaluation included questions related to demographic characteristics, educational level (< 6 years; [6–12] years; ≥ 12 years), living status (alone or not), daily life habits such as alcohol consumption (< 12 g/day; [12;36]; ≥ 36), caffeine consumption (≤ 125 mg/day; [125;375]; > 375), smoking status (none; former; current smoker), and mobility (no restrictions to move; being confined to bed, seat, home, or neighborhood). Health status included body-mass index (BMI) (< 25; [25–30]; ≥ 30 kg/m^2^), hypertension (current antihypertensive treatment or systolic/diastolic blood pressure>160/95 mmHg), diabetes (treatment with antidiabetic agents or fasting glucose level ≥ 7.0 mmol/L), hypercholesterolemia (treatment with lipid-lowering agents or total cholesterol level ≥ 6.2 mmol/L), and cardio-cerebrovascular diseases (angina pectoris, myocardial infarction, cardiovascular surgery, arteritis, and stroke). Depressive status was defined as a score ≥ 16-point on the Center for Epidemiological Studies-Depression Scale or current antidepressant treatment. APOE-ɛ4 was genotyped as described previously [21]. Non-fasting plasma β-amyloid peptide 40 (Aβ_40_) and 42 (Aβ_42_) samples were collected at baseline in a subgroup in tubes containing salt ethylenediaminetetra-acetic as an anticoagulant. Following centrifugation, plasma samples were aliquoted into polypropylene tubes, stored at − 80°C and only thawed immediately prior to β-amyloid quantification. Plasma β-amyloid peptide levels were measured blind to cognitive status. The plasma β-amyloid peptide assay was performed using the INNO-BIA kit (Innogenetics, Ghent, Belgium), based on a multiplex xMAP technique (Luminex, Austin, TX) [22, 23].

### Statistical analyses

Cox models on complete data with delayed entry and age of the participants as the time scale were used to estimate the hazard ratios (HR) and their 95% confidence intervals (CI) for the associations between sleep complaints, hypnotic use, and the incidence of all-cause dementia over the 12-year follow-up. Several multivariable models including study center, age, sex, and potential confounders selected from the literature and associated with all-cause dementia in univariate models (*p* < 0.15) were successively performed. Model 1 was adjusted for study center, age, sex, education, mobility, and APOE-ε4 allele. Model 2 was further adjusted for cardiovascular and metabolic diseases, and model 3 was further adjusted for depressive status. The proportional hazards assumption was tested using the Schoenfeld residuals. In the presence of non-proportionality, stratification on the dependent variable was applied. Incident AD and DVC were further analyzed as distinct end-points. Additional sensitivity analyses were performed to ensure the robustness of the results and to address possible reverse causality: (1) adjustment for hypnotic use, (2) exclusion of participants using hypnotics, (3) imputation of missing data on covariates in 539 participants using Multiple Imputation Chained Equation (7% of missing data, 10 imputation templates were generated), and (4) exclusion of dementia cases at a 2-year follow-up. In supplementary analyses, time-dependent Cox models were performed to take into account the use of hypnotics during the follow-up. The use of hypnotic was considered to be a time-dependent variable, with data for hypnotics use defined throughout follow-up as: no user (no hypnotic during the follow-up), intermittent users (cumulative proportion of hypnotic use ≤ 50% over the entire follow-up), and persistent users (cumulative proportion of hypnotic use > 50% over the entire follow-up). Interactions between significant sleep complaints, hypnotic use, and several factors describing population at higher risk for dementia (i.e., age, sex, depressive status, APOE-ɛ4, history of cardiovascular, and metabolic diseases) on dementia risk were tested using the Wald-*χ*^2^ test for all-cause dementia, AD, and DVC.

In a subsample, Cox models were performed to study the association between plasma Aβ levels and incident dementia. Linear regressions were used to study the association between sleep complaints, hypnotics intake, and β-amyloid levels (taken as a continuous variable or categorized into tertiles). Significance level was set at a two-sided *p* < 0.05. Statistical analyses were performed using SAS 9.4 (SAS Institute Inc.) and R 3.6.3 statistical software.

## Results

### Subjects characteristics

**Fig. 1:**
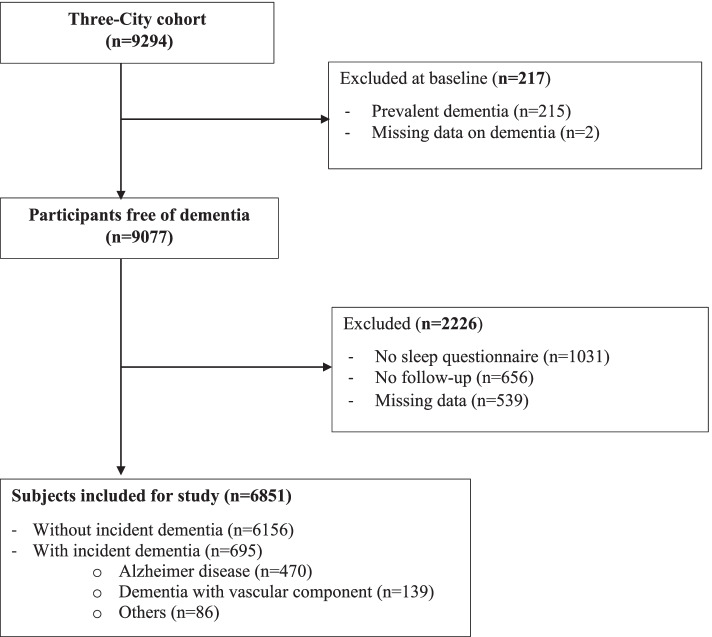
Flow chart of the sample. This figure represents the sample selection for the analysis of excessive daytime sleepiness, insomnia complaints and hypnotic use, and the risk of dementia

As shown in the study diagram (Fig. 1), the study sample included 6851 participants free of dementia (60% women) with a mean age of 73.7 years (SD = 5.3), with completed sleep questionnaires, with follow-up, and no missing data on covariates. Subjects excluded from the study were more frequently female, older, had a lower education level, living alone, and were more often confined, and present more metabolic and cardiovascular diseases, depressive status, and sleep complaints.

Regarding baseline sleep characteristics, the presence of EDS, DIS, DMS, and EMA was reported by 18.1%, 35.3%, 63.7%, and 36.8% of the participants respectively, while the presence of a poor SQ was reported by 52.6% of the participants. Around a quarter (23%) reported no sleep complaints, 25% one complaint, 50% two to four complaints, and only 2% had the five complaints. A higher CSS score was found older adults and in subjects with more cardiovascular diseases and depressive status.

Hypnotics at baseline were taken by 22.5% of subjects: 16.2% BZD, 5.0% BZD-like compounds, 1.9% antihistaminic compounds, 1.4% sedative antidepressants, and 1.2% other medications. Among these subjects treated with hypnotics at baseline, 28.2% had a poor SQ, 21.5% EDS, 57.4% DIS, 70.2% DMS, and 51.3% EMA. Compared to participants without hypnotics use, they were more likely to have increased EDS and insomnia complaints, a poorer SQ, and a higher CSS score (*p* < 0.0001, for all comparisons).

### Associations between sleep complaints, hypnotic intake, and incident dementia over the 12-year follow-up

After a median follow-up of 8.9 years [IQR = 3.9–11.2 years], 695 participants were diagnosed with dementia: 470 with AD, 139 with DVC, and 86 with other dementias. Compared to participants free of dementia, those with incident all-cause dementia had more frequently a lower educational level, diabetes mellitus, cardiovascular disorder, and depressive status and were more likely to be confined at home and carriers of APOE-ε4 (Table 1).

**Table 1:** Baseline demographic and clinical characteristics of 6851 participants according to all-cause dementia over a 12-year follow-up

Variables	Whole sample, *n* = 6851		Free of dementia, *n* = 6156		Incident dementia, *n* = 695		HR [95%CI]	*p* value
	*n*	%	*n*	%	*n*	%		
**Sex**, women	4118	60.11	3672	59.65	446	64.17	1.03 [0.89–1.21]	0.67
**Education (years)**								< 0.0001
< 6	1583	23.11	1356	22.03	227	32.66	1	
[6; 11]	2484	36.26	2276	36.97	208	29.93	0.57 [0.47–0.69]	
≥ 12	2784	40.64	2524	41.00	260	37.41	0.61 [0.51–0.73]	
**Live alone**, yes	2338	34.13	2062	33.50	276	39.71	1.00 [0.85–1.16]	0.97
**BMI (kg/m**^**2**^**)**								0.17
<25	3280	47.88	2959	48.07	321	46.19	1	
[25; 30]	2683	39.19	2399	38.97	284	40.86	1.13 [0.96–1.32]	
≥ 30	888	12.96	798	12.96	90	12.95	1.21 [0.96–1.53]	
**Smoking status**, current/past	2692	39.29	2442	39.67	250	35.97	0.99 [0.85–1.16]	0.92
**Mobility**, confined	352	5.14	279	4.53	73	10.50	1.98 [1.54–2.53]	< 0.0001
**Alcohol (g/day)**								0.45
< 12	4423	64.56	3959	64.31	464	66.76	1	
[12; 36[	1835	26.78	1663	27.01	172	24.75	0.91 [0.77–1.09]	
≥ 36	593	8.66	534	8.67	59	8.49	1.08 [0.83–1.42]	
**Caffeine (mg/day)**								0.79
≤ 125	1767	25.79	1577	25.62	190	27.34	1	
]125; 375]	4029	58.81	3629	58.95	400	57.55	0.95 [0.80–1.13]	
> 375	1055	15.40	950	15.43	105	15.11	1.00 [0.79–1.27]	
**Hypercholesterolemia**, yes	3888	56.75	3483	56.58	405	58.27	1.11 [0.95–1.29]	0.19
**Diabetes mellitus**, yes	613	8.95	516	8.38	97	13.96	1.89 [1.52–2.34]	< 0.0001
**Hypertension**, yes	4080	59.55	3640	59.13	440	63.31	1.06 [0.91–1.24]	0.46
**History of cardiovascular disorders**, yes	1882	27.47	1660	26.97	222	31.94	1.17 [1.00–1.38]	0.05
**Depressive status**^a^, yes	1726	25.19	1482	24.07	244	35.11	1.67 [1.43–1.95]	< 0.0001
**Carrier of the APOE-ε4 allele**, yes	1346	19.65	1151	18.70	195	28.06	1.98 [1.68–2.34]	< 0.0001
**In a subsample of 984 subjects**								
**Aβ**_**40**_ **(pg/ml)**								0.91
< 214.31	328	33.33	295	33.26	33	34.02	1	
[214.31; 251.96]	328	33.33	297	33.48	31	31.96	0.92 [0.56; 1.50]	
> 251.96	328	33.33	295	33.26	33	34.02	0.91 [0.56; 1.47]	
**Aβ**_**42**_ **(pg/ml)**								0.09
< 33.69	326	33.23	292	33.03	34	35.05	1	
[33.69; 42.30]	329	33.54	289	32.69	40	41.24	1.15 [0.73; 1.81]	
> 42.30	326	33.23	303	34.28	23	23.71	0.65 [0.38; 1.10]	
**Ratio Aβ**_**42**_**/Aβ**_**40**_								0.24
< 0.150	330	33.64	293	33.14	37	38.14	1	
[0.150; 0.182]	319	32.52	284	32.13	35	36.08	1.01 [0.63; 1.60]	
> 0.182	332	33.84	307	34.73	25	25.77	0.68 [0.41; 1.12]	

^a^Center for Epidemiological Studies-Depression Scale ≥ 16 or current antidepressant treatment

EDS was associated with incident all-cause dementia even after adjustment for cardiovascular comorbidities (Table 2, model 2) or further adjustment for depressive status (Table 2, model 3). EDS was also associated with incident DVC but no association was observed for AD patients (Tables 3 and 4). No association was found between DIS, DMS, EMA, SQ, and CSS score and either all-cause dementia incidence (Table 2) or the subtypes of dementia (Tables 3 and 4). All these results remained unchanged after adjustment for hypnotic intake or excluding participants taking hypnotics (analyses on 5313 subjects (of whom 332 AD and 96 DVC)). Imputations of the 7% of missing covariates data (Fig. 1) yielded also consistent results with the main analysis (*n* = 7390 (of whom 522 AD and 157 DVC)) as well as excluding incident dementia on the first 2-year follow-up (analyses on 6752 subjects (of whom 403 AD and 119 DVC)).

**Table 2:** Associations between sleep complaints, hypnotics use, and incidence of all-cause dementia over a 12-year follow-up

Variables	Whole sample		Incident dementia				Model 1^a^		Model 2^b^		Model 3^c^	
	*n* = 6851		No, *n* = 6156		Yes, *n* = 695							
	*n*	%	*n*	%	*n*	%	HR [95%CI]^d^	*p*	HR [95%CI]^d^	*p*	HR [95%CI]^d^	*p*
**Excessive daytime sleepiness (EDS)**								0.002		0.007		0.04
No	5610	81.89	5090	82.68	520	74.82	1		1		1	
Yes	1241	18.11	1066	17.32	175	25.18	1.33 [1.11–1.60]		1.29 [1.07–1.55]		1.21 [1.01–1.46]	
**Difficulty with initiating sleep (DIS)**								0.99		0.99		0.30
No	4431	64.68	4018	65.27	413	59.42	1		1		1	
Yes	2420	35.32	2138	34.73	282	40.58	1.00 [0.85–1.18]		1.00 [0.85–1.18]		0.92 [0.78–1.08]	
**Difficulty in maintaining sleep (DMS)**								0.40		0.27		0.10
No	2486	36.29	2247	36.50	239	34.39	1		1		1	
Yes	4365	63.71	3909	63.50	456	65.61	0.93 [0.80–1.10]		0.91 [0.78–1.07]		0.87 [0.75–1.03]	
**Early morning awakening (EMA)**								0.95		0.87		0.47
No	4331	63.22	3921	63.69	410	58.99	1		1		1	
Yes	250	36.78	2235	36.31	285	41.01	1.00 [0.85–1.17]		0.99 [0.84–1.16]		0.94 [0.80–1.11]	
**Sleep quality (SQ)**								0.59		0.49		0.06
Good	2927	47.40	2630	47.46	297	46.85	1		1		1	
Bad	3248	52.60	2911	52.54	337	53.15	0.96 [0.82–1.12]		0.95 [0.81–1.11]		0.85 [0.73–1.00]	
**Clinical severity sleep**^e^ **(CSS)**	6.22 (±3.23)		6.17 (±3.22)		6.63 (±3.32)		1.00 [0.98–1.03]	0.77	1.00 [0.98–1.03]	0.98	0.98 [0.96–1.01]	0.16
**Clinical severity sleep (CSS)**								0.78		0.78		0.27
[0;4]	2071	33.54	1893	34.16	178	28.08	1		1		1	
[4;7.5]	2199	35.61	1961	35.39	238	37.54	1.07 [0.88–1.30]		1.05 [0.86–1.27]		0.99 [0.81–1.20]	
> 7.5	1905	30.85	1687	30.45	218	34.38	1.01 [0.82–1.25]		0.98 [0.79–1.21]		0.86 [0.69–1.06]	
**Hypnotic use**								< 0.0001		< 0.0001		0.001
No	5313	77.55	4833	78.51	480	69.06	1		1		1	
Yes	1538	22.45	1323	21.49	215	30.94	1.45 [1.24–1.72]		1.46 [1.23–1.72]		1.32 [1.11–1.57]	

^a^Adjusted for study center, sex, mobility, and presence of the APOE-ε4 allele, stratified for the level of education

^b^Adjusted for all covariates in model 1 plus diabetes mellitus, body-mass index, and cardiovascular disease

^c^Adjusted for all covariates in model 2 plus depressive status

^d^Estimated using Cox proportional hazard models with delayed entry and age as the time scale

^e^Continuous variables were expressed as mean (standard deviation)

**Table 3:** Associations between sleep complaints, hypnotics use, and incidence of Alzheimer dementia over a 12-year follow-up

Variables	Whole sample		Incident Alzheimer dementia									
	*n*=6626		No, *n* = 6156		Yes, *n* = 470		Model 1^a^		Model 2^b^		Model 3^c^	
	*n*	%	*n*	%	*n*	%	HR [95%CI]^d^	*p*	HR [95%CI]^d^	*p*	HR [95%CI]^d^	*p*
**Excessive daytime sleepiness (EDS)**								0.28		0.33		0.58
No	5457	82.36	5090	82.68	367	78.09	1		1		1	
Yes	1169	17.64	1066	17.32	103	21.91	1.14 [0.90–1.43]		1.12 [0.89–1.42]		1.07 [0.85–1.35]	
**Difficulty with initiating sleep (DIS)**								0.56		0.61		0.22
No	4304	64.96	4018	65.27	286	60.85	1		1		1	
Yes	2322	35.04	2138	34.73	184	39.15	0.94 [0.77–1.15]		0.95 [0.78–1.16]		0.88 [0.72–1.08]	
**Difficulty in maintaining sleep (DMS)**								0.19		0.16		0.07
No	2417	36.48	2247	36.50	170	36.17	1		1		1	
Yes	4209	63.52	3909	63.50	300	63.83	0.88 [0.73–1.06]		0.87 [0.72–1.06]		0.84 [0.69–1.02]	
**Early morning awakening (EMA)**								0.76		0.77		0.50
No	4201	63.40	3921	63.69	280	59.57	1		1		1	
Yes	2425	36.60	2235	36.31	190	40.43	0.97 [0.80–1.18]		0.97 [0.80–1.18]		0.94 [0.77–1.14]	
**Sleep quality (SQ)**								0.99		0.99		0.39
Good	2824	47.30	2630	47.46	194	45.22	1		1		1	
Bad	3146	52.70	2911	52.54	235	54.78	1.00 [0.82–1.22]		1.00 [0.82–1.22]		0.92 [0.75–1.12]	
**Clinical severity sleep**^e^ **(CSS)**	6.20 (± 3.23)		6.17 (± 3.22)		6.57 (± 3.34)		1.00 [0.97–1.03]	0.98	1.00 [0.97–1.03]	0.94	0.98 [0.95–1.01]	0.28
**Clinical severity sleep (CSS)**								0.94		0.93		0.47
[0; 4]	2021	33.85	1893	34.16	128	29.84	1		1		1	
[4; 7.5]	2113	35.39	1961	35.39	152	35.43	0.96 [0.76–1.22]		0.96 [0.76–1.22]		0.91 [0.72–1.16]	
> 7.5	1836	30.75	1687	30.45	149	34.73	0.97 [0.75–1.24]		0.96 [0.75–1.23]		0.85 [0.66–1.10]	
**Hypnotic use**								0.0005		0.002		0.02
No	5165	77.95	4833	78.51	332	70.64	1		1		1	
Yes	1461	22.05	1323	21.49	138	29.36	1.42 [1.17;1.74]		1.38 [1.13–1.69]		1.28 [1.04–1.58]	

^a^Adjusted for study center, sex, mobility, and presence of the APOE-ε4 allele, stratified for the level of education

^b^Adjusted for all covariates in model 1 plus diabetes mellitus, body-mass index, and cardiovascular disease

^c^Adjusted for all covariates in model 2 plus depressive status

^d^Estimated using Cox proportional hazard models with delayed entry and age as the time scale

^e^Continuous variables were expressed as mean (standard deviation)

**Table 4:** Associations between sleep complaints, hypnotics use, and incidence of dementia with vascular component over a 12-year follow-up

Variables	Whole sample		Incident DVC									
	*n* = 6295		No, *n* = 6156		Yes, *n* = 139		Model 1^a^		Model 2^b^		Model 3^c^	
	*n*	%	*n*	%	*n*	%	HR [95%CI]^d^	*p*	HR [95%CI]^d^	*p*	HR [95%CI]^d^	*p*
**Excessive daytime sleepiness (EDS)**								0.001		0.008		0.02
No	5186	82.38	5090	82.68	96	69.06	1		1		1	
Yes	1109	17.62	1066	17.32	43	30.94	1.87 [1.28–2.74]		1.68 [1.15–2.47]		1.58 [1.07–2.32]	
**Difficulty with initiating sleep (DIS)**								0.52		0.63		0.99
No	4098	65.10	4018	65.27	80	57.55	1		1		1	
Yes	2197	34.90	2138	34.73	59	42.45	1.13 [0.78–1.62]		1.09 [0.76–1.58]		1.00 [0.69–1.45]	
**Difficulty in maintaining sleep (DMS)**								0.62		0.98		0.78
No	2290	36.38	2247	36.50	43	30.94	1		1		1	
Yes	4005	63.62	3909	63.50	96	69.06	1.10 [0.76–1.58]		1.01 [0.69–1.45]		0.95 [0.66–1.37]	
**Early morning awakening (EMA)**								0.64		0.56		0.38
No	4006	63.64	3921	63.69	85	61.15	1		1		1	
Yes	2289	36.36	2235	36.31	54	38.85	0.92 [0.64–1.31]		0.90 [0.63–1.29]		0.85 [0.60–1.22]	
**Sleep quality (SQ)**								0.37		0.27		0.08
Good	2692	47.53	2630	47.46	62	50.41	1		1		1	
Bad	2972	52.47	2911	52.54	61	49.59	0.85 [0.59–1.21]		0.82 [0.57–1.17]		0.72 [0.50–1.04]	
**Clinical severity sleep**^e^ **(CSS)**	6.18 (±3.22)		6.17 (±3.22)		6.53 (±3.05)		0.99 [0.94–1.05]	0.81	0.98 [0.92–1.04]	0.46	0.96 [0.90–1.02]	0.15
**Clinical severity sleep (CSS)**								0.06		0.09		0.06
[0; 4]	1922	33.93	1893	34.16	29	23.58	1		1		1	
[4; 7.5]	2019	35.65	1961	35.39	58	47.15	1.58 [1.00–2.48]		1.41 [0.89–2.22]		1.30 [0.83–2.06]	
> 7.5	1723	30.42	1687	30.45	36	29.27	1.03 [0.62–1.72]		0.91 [0.55–1.52]		0.79 [0.47–1.33]	
**Hypnotic use**								0.02		0.04		0.26
No	4929	78.30	4833	78.51	96	69.06	1		1		1	
Yes	1366	21.70	1323	21.49	43	30.94	1.52 [1.06; 2.18]		1.46 [1.01–2.11]		1.24 [0.85–1.81]	

^a^Adjusted for study center, sex, mobility, and presence of the APOE-ε4 allele, stratified for the level of education

^b^Adjusted for all covariates in model 1 plus diabetes mellitus, body-mass index, and cardiovascular disease

^c^Adjusted for all covariates in model 2 plus depressive status

^d^Estimated using Cox proportional hazard models with delayed entry and age as the time scale

^e^Continuous variables were expressed as mean (standard deviation)

The use of hypnotics at baseline, particularly BZD (72% of sleep medication), was independently associated with a higher risk of all-cause dementia, AD, and DVC even after several adjustments (Tables 2, 3, and 4, model 2). When further adjusting for depressive status, the association was only significant for all-cause dementia and AD (Tables 2, 3, and 4, model 3).

In supplementary analyses, we examined the association between persistent use of hypnotics and the risk of dementia in two different ways. First, we studied the relationship between the use of hypnotics during the first 2 years and the risk of dementia. A total of 4454 subjects (68.3%) did not report hypnotic use at baseline and at 2 years of follow-up, 1203 (18.5%) reported using both at baseline and at 2 years (persistent users), and 865 (13.3%) were taking hypnotics at one of two time points (intermittent users). The risk of all-cause dementia for the next 10 years was associated with the persistent use of hypnotics (p-global=0.009, compared to non-users, HR = 1.20, 95%CI = [0.94–1.53] for intermittent users; HR = 1.37, 95%CI = [1.12–1.68] for persistent users; model 3). Similar results were found for AD (p-global = 0.04, compared to non-users, HR = 1.10, 95%CI = [0.81–1.49] for intermittent users; HR = 1.39, 95%CI = [1.08–1.79] for persistent users). No association was found between the use of hypnotics and incident DVC. Second, we reported the use of hypnotics throughout the follow-up, with 4029 (58.8%) non-user, 1488 (21.7%) persistent users, and 1334 (19.5%) intermittent users. Compared to non-user, persistent users were associated with dementia (HR = 2.15, 95%CI = [1.76–2.63]; model 3) while there was no association for intermittent users (HR = 0.99, 95%CI = [0.82–1.19]; model 3).

### Interaction between EDS, hypnotic intake, and factors describing specific populations with regard to dementia incidence

Association between hypnotic use at baseline and incident all-cause dementia differed according to diabetes status (*p* = 0.03 for interaction test) with a higher risk in diabetic subjects (HR = 2.22, 95%CI = [1.47–3.37]) compared to non-diabetics (HR = 1.36, 95%CI = [1.13–1.62]). Similar results were found for incident AD but not DVC. No interaction was found between EDS, hypnotic use, and factors describing specific populations (i.e., sex, depressive status, APOE-ɛ4, history of metabolic, and cardiovascular diseases) on the risk of all-cause dementia and subtypes.

The sample was also stratified according to age (≥ 75 years (median of the whole sample) vs < 75 years). EDS was associated with all-cause dementia incidence in the older group after adjustments (HR = 1.29, 95%CI = [1.02–1.64]) but not in the youngest group (HR = 1.11, 95%CI = [0.82–1.49]). Results were unchanged for DVC, but no association was found between EDS and AD in any age group. The use of hypnotics at baseline was associated with all-cause dementia incidence in both age groups and with AD only in those under 75 years of age.

### Associations between sleep complaints, hypnotics, plasma β-amyloid levels, and incident dementia

These analyses were implemented on a subgroup of 984 subjects with data available for plasma Aβ measurements. These subjects were not different from the 5867 without these measurements: 60% of women, mean age of 73.6 (± 5.2), 18.7% complained of EDS, 36.1% of DIS, 64.9% of DMS, 36.2% of EMA, and 22.7% were taking hypnotics at baseline and 9.9% were diagnosed dementia over the follow-up.

No association was found between baseline plasma Aβ_42_, Aβ_40_, and its ratio (taken continuously or categorized into tertile) and (1) sleep complaints taken alone or combined, and hypnotic use at baseline, (2) and incident all-cause dementia and its subtypes.

## Discussion

In this large cohort of community-dwelling elderly, EDS was associated with an increased risk of 21% for incident all-cause dementia and 58% for DVC over 12 years, but no association was observed with AD incidence. Persistent use of hypnotics increased the risk for all-cause dementia and AD, but not DVC after adjustment for the depressive status. In contrast, no association was found between insomnia complaints, and SQ taken as individual factors or combined with EDS in the CSS score on the risk of incident dementia. We also reported that the association between hypnotic use and incident all-cause dementia and AD was stronger in diabetic subjects, with same trend in subjects below 75 years old for incident AD. The association between EDS and all-cause dementia and DVC risk appeared greater in subjects over 75 years old.

EDS is a frequent condition in the elderly population. It may be due to multifactorial factors and may be associated with severe consequences [24, 25]. EDS is often comorbid with depression, poor sleep habits, obesity, cardiovascular disease, obstructive sleep apnea (OSA), and hypnotics intake, each of which being also associated with an increased risk of cognitive impairment [1, 26–28]. Prospective studies in older adults reported that EDS was independently associated with cognitive decline and dementia [2, 3, 29]. Our current results confirm and extend this finding in both all-cause dementia and DVC, within a larger sample of both men and women elderly persons over a long follow-up period, and with further adjustments particularly APOE-ε4 status, cardiovascular, and metabolic diseases and depression. The mechanism underlying this association remains unclear. Self-reported EDS may be interpreted as a marker of sleep instability, with fragmented sleep and reduced slow-wave sleep, but also with OSA especially when it is severe. Recent data suggested that excessively sleepy OSA subtype has the greatest increased cardiovascular risk compared to other types of OSA [30]. Elderly subjects may lack of awareness of sleep apnea symptoms, but sleep apnea is associated with a higher risk of dementia and cardiovascular disorders [27, 31]. Several pathophysiological mechanisms may be involved such as disturbed slow-wave and rapid eye movement sleep, intermittent hypoxia, low-grade inflammation, oxidative stress, and brain Aβ aggregation and tau hyperphosphorylation [32, 33]. Other studies also underlined the association between EDS, but not insomnia complaints, and higher risk of cardiovascular events [34, 35], the latter being potential mediators in the relationship between EDS and DVC incidence [3]. Here, we found that subjects with EDS over 75 years of age exhibited the highest risk of dementia, suggesting that EDS would be a predictor for late-onset age of dementia.

EDS in non-demented elderly subjects could also be associated with underlying neurodegeneration. A recent study found an association between EDS and global cortical thickness reduction primarily in regions with increased age-susceptibility which may indicate accelerated brain aging [36]. Degeneration of wakefulness-promoting neurons in the cholinergic basal forebrain, noradrenergic locus coeruleus, and orexin hypothalamic systems may be damaged and cause sleep/wake abnormalities [37]. Thus, there are biologically plausible mechanisms that may explain these associations; however, further studies are required to explore these hypotheses.

Insomnia is one of the most prevalent sleep complaints in the elderly. We found no association between poor SQ, insomnia complaints and all-cause dementia, AD and DVC. Our results are in agreement with several studies reporting no association [2, 12, 38], but contrast with others showing the association between insomnia and dementia [6, 8, 10]. These inconsistencies across studies may be attributed to differences in methodology (adjustments, follow-up duration) and heterogeneity in cognitive and sleep assessment. Most of the positive associations between insomnia and dementia were not controlled for APOE-ε4, depressive status, and the use of hypnotic [6–8, 10, 33]. To our knowledge, no previous studies evaluated the presence and severity of both daytime and nighttime sleep complaints in the elderly to assess for the potential additive role of sleep problems in the onset of dementia. Although not validated, we proposed to combine in the present study the five sleep complaints (EDS, insomnia complaints, and sleep quality) together in a global assessment called CSS; however, we found no association between this score and the onset of dementia risk.

Hypnotics (mainly BZD) at baseline were taken by almost a quarter of the subjects, who had more frequently EDS, insomnia complaints, and higher CSS score. Hypnotics use increased the risk of all-cause dementia and AD after several adjustments including depressive status. Moreover, hypnotic intake (especially for persistent users) was associated with incident all-cause dementia and AD in subjects below 75 years old and particularly in diabetic subjects. Few studies already underlined that AD and diabetes may share common pathophysiology, with prolonged excess of glucose leading to poor sleep quality and risk of neurodegeneration [39, 40]. The association between BZD use and dementia incidence was also reported [22, 41]; however, the biological mechanism that underlines this association is not well understood. Sleep is critical for ensuring brain metabolic homeostasis with large increases in the cortical interstitial space during slow-wave sleep compared to wake resulting in convective exchange between the cerebrospinal fluid (CSF) and the interstitial fluid [42]. The restorative function of sleep may therefore relate to increased clearance of potentially neurotoxic degradation products of neuronal activity that accumulate in the awake brain, such as Aβ. With the chronic use of BZDs, the delta oscillations decrease, the glymphatic system dedicated to CSF transport, and metabolic waste drainage from the brain may be impaired resulting in Aβ accumulation and deposition [43]. We report here no association between plasma Aβ_40_-Aβ_42_ levels and sleep complaints, with measurements performed at baseline therefore in subjects without dementia. These results were in line with a previous study including only men [8]. However, both studies measured Aβ levels in the plasma, being less sensitive than CSF to estimate the impact of sleep disturbance on Aβ kinetics. An increased longitudinal brain Aβ accumulation assessed on a positron emission tomography was recently reported in elderly subjects with EDS and without dementia [44]. A proposed hypothesis suggests that EDS is a clinical manifestation of increased synaptic or network overload in the cingulate and precuneus regions, areas with high metabolic activity, and Aβ accumulation that may increase oxidative stress and neuronal death, and then the risk of AD.

The strengths of this study were the longitudinal design with a 12-year-follow-up, the large sample size, the low attrition rate, and the large number of potential predictors including socio-demographic, lifestyle, health characteristics, medications, and APOE-ε4 status.

### Limitations

This study had some limitations. Bias could have been introduced by the non-random exclusion of participants with missing data at baseline, who were older, had more comorbidities and sleep complaints and used more hypnotics; this may have underestimated the strengths of the associations reported. Although the prevalent demented subjects were excluded in this study, a potential reverse causality effect cannot be excluded. This phenomenon is inevitable in the study of subjects aged 65 and over because of the prodromal phase onset 10 to 20 years before symptoms. However, after the exclusion of incident demented subjects during the first 2 years of follow-up, the results remained unchanged. The possibility of over-adjustment could not be excluded: potential confounders with cardiovascular parameters could be intermediate variables in the pathway between sleep complaints, hypnotic intake, and dementia incident. Due to the exploratory nature of this study, correction for multiple comparisons were not made. Furthermore, the assessment of sleep complaints was only self-reported causing possible recall bias and a lack of accuracy in responses, although previous studies have assessed EDS with a similar question [29, 34, 35]; however, validated measures such as the Epworth Sleepiness Scale were not available in this study. In addition, we developed for the present study a clinical sleep severity (CSS) score to estimate the potential additive role of the five key sleep complaints on the risk of dementia; however, this CSS was only proposed for this present study and therefore has not been validated by other studies.

The drugs intake was verified by examining the prescriptions and medications themselves; however, duration, frequency, dose, and reason of the medication intake were not recorded. Regarding the duration of exposure to hypnotics, we performed 2 sensitivity analyses. First, we limited the study of the association between the use of hypnotics during the first 2 years and incident of dementia while maintaining a significant follow-up period (10 years). Then, we used time-dependent variable to consider hypnotics use throughout the follow-up. Both analyses showed that persistent use of hypnotic was associated with a higher risk of dementia. We were unable to directly measure symptoms of anxiety in this study. However, our models were adjusted for the CES-D score first designed to screen for depressive symptoms but also anxiety symptoms in the elderly population [45, 46]. The presence of sleep apnea was not evaluated; thus, the confounding effect of an underlying OSA syndrome in the relationship between EDS and the risk of dementia cannot be excluded. Unfortunately, sleep duration and objective measures of sleep and daytime sleepiness were not available for this study. Even difficult within the context of a large epidemiological studies, further studies using objective sleep tools are recommended. Finally, β-amyloid concentrations were assessed in the plasma instead of cerebrospinal fluid, being a less sensitive method, but still relevant to indicate individuals at short-term risk of dementia or cognitive decline [22, 23].

## Conclusions

EDS was associated with a high risk of all-cause dementia and even more strongly with DVC, but not with AD while the use of hypnotics was associated with incident all-cause dementia and AD. These results suggest that EDS and hypnotics intake may be targets for intervention strategies in dementia prevention. No association was found for insomnia complaints and quality of sleep assessed alone or combined with EDS after controlling for potential confounders. Further studies are required to elucidate the biological mechanisms involved in these associations and whether its management can prevent the risk of dementia.

## Data Availability

The datasets used and analyzed during the current study are available from the corresponding author on reasonable request.

## Abbreviations

AD: Alzheimer’s disease
APOE: Apolipropotein-e
BMI: Body mass index
BZD: Benzodiazepine
CI: Confidence intervals
CSF: Cerebrospinal fluid
CSS: Clinical sleep score
DIS: Difficulty in initiating sleep
DMS: Difficulty maintaining sleep
DVC: Dementia with vascular component
EDS: Excessive daytime sleepiness
EMA: Early morning awakening
HR: Hazard ratio
OSA: Obstructive sleep apnea
SQ: Sleep quality
